# Mitochondrial Dysfunction Accounts for the Stochastic Heterogeneity in Telomere-Dependent Senescence 

**DOI:** 10.1371/journal.pbio.0050110

**Published:** 2007-05-01

**Authors:** João F Passos, Gabriele Saretzki, Shaheda Ahmed, Glyn Nelson, Torsten Richter, Heiko Peters, Ilka Wappler, Matthew J Birket, Graham Harold, Karin Schaeuble, Mark A Birch-Machin, Thomas B. L Kirkwood, Thomas von Zglinicki

**Affiliations:** 1 Henry Wellcome Laboratory for Biogerontology Research, Institute for Ageing and Health, University of Newcastle, Newcastle upon Tyne, United Kingdom; 2 Center for Integrated Systems Biology of Ageing and Nutrition, University of Newcastle, Newcastle upon Tyne, United Kingdom; 3 Crucible Laboratory, Life Knowledge Park, University of Newcastle, Newcastle upon Tyne, United Kingdom; 4 Institute of Human Genetics, International Centre for Life, University of Newcastle, Newcastle upon Tyne, United Kingdom; 5 School of Clinical and Laboratory Sciences, University of Newcastle, Newcastle upon Tyne, United Kingdom; Rockefeller University, United States of America

## Abstract

Aging is an inherently stochastic process, and its hallmark is heterogeneity between organisms, cell types, and clonal populations, even in identical environments. The replicative lifespan of primary human cells is telomere dependent; however, its heterogeneity is not understood. We show that mitochondrial superoxide production increases with replicative age in human fibroblasts despite an adaptive UCP-2–dependent mitochondrial uncoupling. This mitochondrial dysfunction is accompanied by compromised [Ca^2+^]_i_ homeostasis and other indicators of a retrograde response in senescent cells. Replicative senescence of human fibroblasts is delayed by mild mitochondrial uncoupling. Uncoupling reduces mitochondrial superoxide generation, slows down telomere shortening, and delays formation of telomeric γ-H2A.X foci. This indicates mitochondrial production of reactive oxygen species (ROS) as one of the causes of replicative senescence. By sorting early senescent (SES) cells from young proliferating fibroblast cultures, we show that SES cells have higher ROS levels, dysfunctional mitochondria, shorter telomeres, and telomeric γ-H2A.X foci. We propose that mitochondrial ROS is a major determinant of telomere-dependent senescence at the single-cell level that is responsible for cell-to-cell variation in replicative lifespan.

## Author Summary

After a limited number of cell divisions, somatic cells lose the capacity for proliferation, called cellular replicative senescence. Senescence, which is triggered by the loss of DNA sequences at the ends of chromosomes (telomeres), is often seen as an example of a regular “biological clock.” However, cell senescence is heterogeneous, with large differences in lifespan between individual cell lineages. This heterogeneity is clearly related to stress, specifically oxidative stress. It was not known, however, whether stress-induced “premature” senescence involves telomeres or is caused by telomere-independent DNA damage responses. Mitochondria are the most important source of reactive oxygen species (ROS) in cells under physiological conditions. We found that mitochondrial function deteriorated while cells approached senescence, leading to increased ROS production. Delaying mitochondrial dysfunction led to postponed replicative senescence and slowing of telomere shortening. Prematurely senescing cells sorted out of young cultures displayed mitochondrial dysfunction, increased oxidative stress, and short telomeres. We propose that replicative telomere-dependent senescence is not “clocked,” but rather is a stochastic process triggered largely by random mitochondrial dysfunction.

## Abstract 3

Mitochondrial uncoupling is used to study the relationship between telomere length, the production of reactive oxygen species, and replicative senescence.

## Introduction

Central to current understanding of biological aging is the idea that limited evolutionary investment in mechanisms of cellular and molecular maintenance and repair causes a gradual accumulation of cellular damage, which in turn causes age-related frailty, disease, and death [1]. It follows that aging is not itself genetically programmed, although longevity is, through the genetic regulation of repair mechanisms. It also follows that multiple molecular mechanisms are expected to participate in cell aging and that the actions of these mechanisms are inherently stochastic, i.e., subject to the variations of chance. The essential multiplicity of mechanisms of aging is widely acknowledged, although in practice, it has not yet been much studied, whereas the stochastic nature of aging is evident in the pronounced variability that is a hallmark of the aging process [2–5]. In this paper, we describe a study of the multiplicity of mechanisms contributing to one of the best-studied cellular models of aging, i.e., the replicative senescence (loss of proliferative capacity) of human diploid fibroblasts (HDFs), which occurs after a number of population doublings in vitro [6]. We show that interactions between mechanisms whose connections have not been previously addressed in this system provide new insights into the underlying biology of replicative senescence. We also show that these interactions explain the marked cell-to-cell variation in replicative lifespan within a culture, which is seen even if cultures are grown clonally from single cell founders [7].

Replicative senescence in HDF is known to be caused ultimately by a DNA-damage response that is triggered by uncapped telomeres [8,9], which in turn result from replication-driven loss of telomere repeats in the absence of telomerase. This process is sometimes interpreted as “programmed aging” at the cell level, although the idea of an underlying program driving replicative senescence is hard to reconcile with its extensive, intrinsic heterogeneity. Furthermore, telomere-shortening rate and cell replicative lifespans can be greatly modified by DNA-damaging oxidative stress [10] via a telomere-specific repair deficiency, which causes stress-dependent accumulation of single-strand breaks [11] and accelerates telomere shortening during DNA replication [12]. This had led to the suggestion that telomere reduction is not strictly programmed, with telomere length acting as a mere cell-division counting device, but instead that telomeres act as sentinels for cumulative oxidative and/or environmental stress triggering division arrest when the damage burden (detected through telomere length) becomes too great [10]. Such an idea is fully consistent with the suggestion that replicative senescence serves a pleiotropic role in aging as an anti-cancer mechanism [13] for which there is growing evidence [14–17], since the damage that triggers senescence would otherwise pose an increased risk of malignancy. It is also consistent with recent evidence linking environmental stress to telomere length in humans [18], which could provide important insights into the nature of the connection between replicative senescence and organismal aging, as well as into the potential malleability of these processes.

It has been recognized for some time that the heterogeneity in telomere-driven replicative senescence challenged the simple idea that in telomerase-negative cells, the telomeres shorten progressively through failure of complete chromosome-end replication. It has been suggested [19] that telomere uncapping, the process that leads to telomeres being recognized as inducers of a DNA-damage response triggering growth arrest, is itself stochastic, but such an effect does not appear sufficient to explain the observed heterogeneity in division capacity within cultures. Rubelj and Vondracek proposed a hypothetical model of “abrupt telomere shortening,” but this amounted only to speculation embodied in equations [20]. A data-driven modelling approach has shown how the effects of oxidative stress on telomere shortening can be used to study the dynamics of replicative senescence in silico and, in particular, how damage-induced mitochondrial defects might underlie stochastic cell-to-cell variation in oxidative stress, which by acting on telomeres can account for the observed heterogeneity [21–23]. Most of the evidence to date for mitochondrial involvement in aging has come from post-mitotic cells, although recently it was shown that extensive, stochastic variability in the presence and level of mitochondrial DNA mutations occurs in human colon stem cells, one of the most actively proliferating cell types in the body [24]. Motivated by this background of theoretical and experimental work, we have examined the possible role of mitochondria and oxidative stress in telomere-driven replicative senescence, something which until now has been unclear [25].

We first showed that various indices of mitochondrial function revealed significant mitochondrial dysfunction in senescent fibroblast cultures as well as a global change in gene expression pattern indicative of enhanced retrograde signalling. The retrograde response includes down-regulation of mitochondrial membrane potential by reactive oxygen species (ROS)-dependent expression of the uncoupling protein UCP-2 and is thought to be at least partly a mechanism of adaptation to high cellular levels of ROS. We then tested whether mitochondrial ROS production plays a causal role in senescence by uncoupling of mitochondria in live cells with 2,4-dinitrophenol. This reduced mitochondrial superoxide generation, slowed the rate of telomere shortening, and delayed formation of telomeric DNA-damage foci containing activated H2A.X, thus extending replicative lifespan. These results indicate that mitochondrial superoxide production is a determinant of telomere-dependent replicative senescence. Finally, we examined whether cell-to-cell heterogeneity of mitochondrial ROS production might account for the variability of cell division capacity. We found that those cells in an actively growing culture that showed a senescent phenotype in terms of morphology and lipofuscin content and stained positive for senescence-associated β-galactosidase (Sen-β-Gal) and γ-H2A.X were similar to cells in a senescent culture in terms of mitochondrial dysfunction, short telomere length, and co-localisation between γ-H2A.X and telomeres.

Our studies confirm an intimate connection in the normal replicative senescence of HDF between oxidative stress and telomere-dependent effects on cell proliferation. They suggest that mitochondrial dysfunction largely determines the age-related development of the extensive cell-to-cell variation in cell division potential.

## Results

### Mitochondrial Dysfunction in Senescent Fibroblasts

Production of ROS, especially superoxide, is a necessary by-product of mitochondrial respiration. Under many conditions, mitochondrial superoxide production is positively coupled to the potential energy for protons ΔΨ across the inner mitochondrial membrane and the mitochondrial membrane potential [26]. Mitochondrial dysfunction is characterized by concurrent high superoxide production and breakdown of membrane potential, often associated with mitochondrial DNA (mtDNA) damage. To assess whether mitochondrial dysfunction is associated with human fibroblast senescence, we compared young and senescent cultures of MRC5 fibroblasts. Young cultures were defined as actively growing (>0.25 population doublings [PD]/day) and containing less than 10% of cells positive for markers of senescence and DNA double-strand breaks (Sen-β-Gal and γ-H2A.X). These conditions were met for PDs up to about 40. Senescent cultures were defined by a net growth rate below 0.025 PD/day and more than 50% of the cells positive for Sen-β-gal and γ-H2A.X staining.

**Figure 1:**
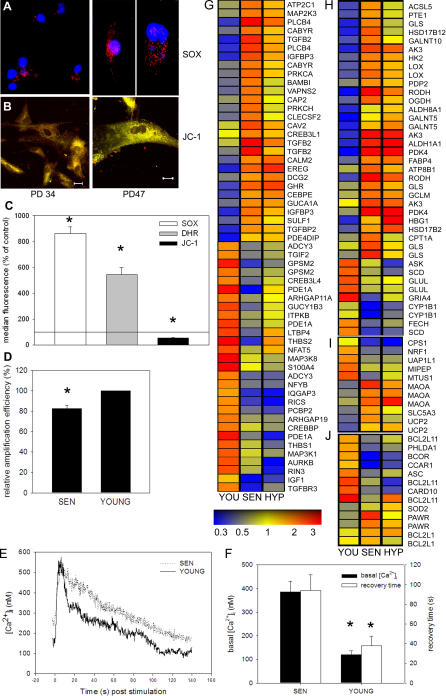
Mitochondrial Dysfunction and Retrograde Response in Senescent Cells (A) and (B) Confocal images of actively proliferating (PD 34) and senescent (PD 47) MRC-5 fibroblasts. (A) Red MitoSOX fluorescence indicates mitochondrial superoxide. Blue: DAPI. Note lower average red fluorescence and cell-to-cell heterogeneity in proliferating cultures. (B) JC-1 ratio images. A shift from red to green fluorescence indicates low MMP. (C) MitoSOX, DHR, and JC-1 ratio intensities in senescent MRC-5 fibroblasts as measured by FACS (percentage of young fibroblast control, set to 100%). Data are mean ± s.e.m., *n* = 3. An asterisk (*) indicates a significant difference to young controls with *p* < 0.001. (D) Integrity of mtDNA as measured by the relative amplification efficiency for an 11-kb mtDNA fragment in young and senescent MRC5 cells. Data are mean ± s.e.m. An asterisk (*) indicates a significant difference to young controls with *p* < 0.001. (E) Single cell traces of cytoplasmic calcium levels [Ca^2+^]_i_ following a challenge with 100 mM CaCl_2_ as measured by Fluo3 fluorescence confocal imaging. (F) Basal cytoplasmic [Ca^2+^]_i_ levels and recovery times in young and senescent cells. Data are mean ± s.e.m. from 13 young and 8 senescent cells. An asterisk (*) indicates a significant difference between young and senescent cells with *p* < 0.001. (G–J) Relative mRNA expression levels in young (YOU), senescent (SEN), and hyperoxia-treated (HYP) MRC5 (averages of two [HYP] to four [YOU] independent experiments per condition). Fold-change colour code is indicated on the bottom. For some genes, the arrays contained probe sets resulting in multiple occurrences. All genes showing at least a 2-fold expression difference between YOU and SEN were selected if the expression difference was confirmed in HYP and if they fell into one of the following categories: (G) Ca-dependent signalling; (H) glycolysis and Krebs cycle; (I) mitochondrial function and biogenesis; and (J) stress response and apoptosis.

Using a fluorescent probe that specifically detects superoxide within the mitochondrial matrix (MitoSOX; Figure 1A), we found that mitochondria in senescent fibroblasts produced more superoxide than young ones (fluorescence increase by 863% ± 49% *p* = 0.003) (Figure 1C). Moreover, cellular peroxides as measured by staining with dihydrorhodamine 123 (DHR) were increased in senescence (fluorescence increase by 545% ± 52%, *p* = 0.01) (Figure 1C). Surprisingly, the mitochondrial membrane potential (MMP), measured as JC-1 fluorescence ratio by confocal microscopy (Figure 1B) and by flow cytometry (Figure 1C), was decreased (fluorescence ratio decrease to 54.6% ± 4.1%, *p* = 0.008).

There was an increase in mitochondrial mass and mitochondrial DNA copy number in senescent cells (Figure S1); however, the increase in MitoSOX and DHR fluorescence levels remained significant even when expressed per unit of mitochondrial mass (unpublished data). Increased mitochondrial ROS production, despite decreased MMP, indicated significant mitochondrial dysfunction in senescent cells. This is in accordance with increased mtDNA damage in senescent cells as shown by decreased amplification efficiency for a large (11 kilobase [kb]) fragment of mtDNA (Figure 1D; see Protocol S1 for measurement of mtDNA damage).

### Retrograde Response in Senescent Fibroblasts

Mitochondrial dysfunction induces a major reprogramming of nuclear gene expression, termed the retrograde response [27], which is a feature of replicative aging in budding yeast [28] and can be induced by severe mtDNA depletion in human cells [29]. Retrograde response includes dysregulation of Ca^2+^-dependent signalling, up-regulation of mitochondrial biogenesis [30], and major metabolic and anti-apoptotic adjustments [27]. However, induction of retrograde signalling in mammalian cellular senescence has not been demonstrated so far.

An essential trigger for retrograde response is believed to be the increased cytoplasmic [Ca^2+^]_i_ levels due to low Ca^2+^ storage capacity of dysfunctional mitochondria with low membrane potential [27]. Senescent fibroblasts displayed significantly higher cytoplasmic Ca^2+^ levels under basal conditions as well as slower recovery after a Ca challenge as measured by live cell confocal imaging using the Ca^2+^-sensitive dye Fluo3 (Figure 1E and 1F). Senescent fibroblasts also show increased mitochondrial mass and number (Figure S1) consistent with activation of mitochondrial biogenesis [30].

To further characterize retrograde response in fibroblast senescence, we performed an RNA microarray expression analysis (see Protocol S1). KEGG pathway analysis comparing young with senescent cells indicated significant enrichment of differentially expressed genes in various major Ca^2+^-related signalling pathways as well as in energy (nitrogen and sulphur), nucleotide, and amino acid metabolism (Table S2). Expression of 2,610 gene spots changed at least 2-fold between young and senescent cells, with the majority of these (1,522) down-regulated in senescence as compared to young fibroblasts (Figure S2). A limited number of these changes (mostly up-regulations) were confirmed in cells that reached senescence under hyperoxia (Figure S2). From all genes that were changed similarly under both normoxic and hyperoxic senescence, 92 genes (120 probe sets) could be assigned to one of four processes that are known to be activated in retrograde response: Ca^2+^-binding and Ca^2+^-mediated signalling (Figure 1G), glycolysis and Krebs cycle enzymes (Figure 1H), mitochondrial biogenesis and function (Figure 1I), and stress response (Figure 1J). The majority of these genes (69) are up-regulated in senescence.

In particular, we noted activation of enzymes involved in calcium/calmodulin-dependent signalling, including PRKC A and H, PLCB4, EREG, CEBPE, CREB3L1 and -L4, TGFβ2, and IGFBP3 (Figure 1G). A number of enzymes involved in glucose metabolism, such as HK2, PDP2, and PDK4 (Figure 1H), glutamate metabolism, such as GCLM and GLUL (Figure 1H), and lipid metabolism, such as CPT1A, PTE1, and FABP4 (Figure 1H), were up-regulated, as well as enzymes with major functions in mitochondrial metabolism, such as AK3, PDK4 (Figure 1H), and MAOA (Figure 1I). Up-regulation of some of these genes in senescence and under hyperoxia was confirmed by reverse-transcriptase (RT)-PCR (Figure S3).

Increased resistance of cells to stress and apoptosis has also been described as part of the retrograde response. We observed increased mRNA expression of the antioxidant SOD2 and the anti-apoptotic BCL-2 family member BCL2L1 (BCL-X), and a decrease in the expression of the pro-apoptotic BCL2L11 (BIM) (Figure 1J).

Taken together, the fact that mitochondrial dysfunction during senescence of human MRC5 fibroblasts is accompanied by increased mitochondrial biogenesis, compromised [Ca^2+^]_i_ handling, and transcriptional up-regulation of genes involved in Ca-mediated signalling, stress response, glycolysis, and mitochondrial function suggests that cellular senescence might be associated with the induction of retrograde response, which in turn is likely to be caused by mitochondrial dysfunction.

### Cellular ROS Levels Control MMP via UCP-2 Expression

The finding that low MMP was associated with high mitochondrial superoxide levels in senescent cells was unexpected, because it is well established that lowering MMP (and thus ΔΨ, allowing leakage of protons across the inner mitochondrial membrane) decreases mitochondrial ROS production in isolated mitochondria [31]. However, it is also known that ROS and ROS-derived reactive aldehydes activate uncoupling proteins and can thus lower MMP [32].

**Figure 2:**
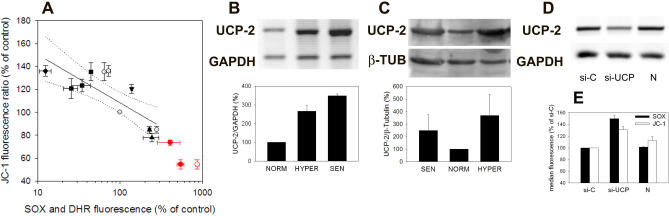
Cellular ROS Levels Control MMP via UCP-2 Expression (A) Interventions that modify ROS result in an inverse change in MMP. Fibroblasts were grown under 5% (squares) or 40% (triangles) oxygen. Cells were also treated with the ROS scavenger PBN (cross-haired square and inverted triangle) or transfected with an SOD3 transgene [34] (diamond). Open symbols: MitoSOX, filled symbols: DHR. Controls (circles) were set as 100% for each experiment. Data are mean ± s.e.m from three to five experiments per treatment. Solid line: linear regression, dotted lines: 95% confidence intervals. Red symbols: senescent and SES MRC-5 fibroblasts. (B) UCP-2 expression in MRC-5. Top: Duplex-RT-PCR of UCP-2 versus GAPDH. Bottom: quantification of results, *n* = 2, mean + difference between means (Δm). NORM indicates young controls proliferating under normoxia, HYPER indicates young cells under 40% hyperoxia, and SEN indicates senescent cells under normoxia. (C) UCP-2 versus β-tubulin (β-TUB) protein content. Top: Immunoblots; bottom: quantification of results. Data are mean + Δm, *n* = 2. (D) Suppression of UCP-2 expression in MRC5 fibroblasts by treatment with siRNA (si1-UCP), but not with a scrambled control siRNA (si-C) as measured by RT-PCR. N indicates untreated control. (E) si1-UCP expression results in increased mitochondrial superoxide levels (SOX) and increased MMP (JC-1 ratio). Data are mean + Δm, *n* = 2. Similar results were obtained with si2-UCP and si3-UCP.

To test whether MMP was determined by cellular ROS levels in human fibroblasts, we first manipulated ROS levels over about one order of magnitude. Cellular and mitochondrial ROS levels increased 2–3-fold when young human fibroblasts were grown under increased oxygen partial pressure (40%) for 1 wk. Treatment with the free radical scavenger α-phenyl N-tert butylnitrone (PBN) blocked this increase. Low ambient oxygen (5%), treatment with PBN under normoxia [33], or overexpression of the human *SOD3* gene [34] all resulted in a 2–5-fold decrease of ROS levels (Figure 2A). MMP correlated inversely with both mitochondrial superoxide (*n* = 4, *r* ^2^ = 0.710) and cellular peroxide levels (*n* = 8, *r* ^2^ = 0.812, Figure 2A), resulting in *p* = 0.0005 (analysis of variance [ANOVA]) for the overall correlation. The same correlation was predictive for MMP levels in senescent and sorted early senescent (SES) MRC-5 cells, which had high ROS levels (Figure 2A).

Within the inner mitochondrial membrane, there is a family of anion carriers which, if expressed, uncouple mitochondria by allowing proton leakage across the membrane. This family includes the uncoupling proteins UCP-1, UCP-2, and UCP-3, and the adenine nucleotide translocase (SLC25A5A). We found that UCP-2 expression was increased at the mRNA level in cells that senesced under either normoxia or hyperoxia (Figure 1J). Up-regulation of UCP-2 in senescent cells was confirmed by RT-PCR (Figure 2B) and Western blotting (Figure 2C).

To test whether UCP-2 regulated MMP and mitochondrial superoxide production in intact human fibroblasts, we knocked down UCP-2 by transient transfection with small interfering RNAs (siRNAs). Reducing UCP-2 mRNA levels by about 50% resulted in an enhanced MMP, as measured by a 31% ± 6% (mean ± the standard deviation [SD], *p* = 0.016) increase of the JC-1 fluorescence ratio and in a 50% ± 5% (*p* = 0.006) increase in MitoSOX fluorescence (Figure 2D).

### Mitochondrial Uncoupling Delays Telomere-Dependent Senescence

There is good evidence that reducing cellular ROS levels can extend the replicative lifespan of human fibroblasts [35,36], and delay telomere shortening [33,34,37,38], suggesting that mitochondrial dysfunction contributes to replicative senescence. However, other data indicate that mitochondrial ROS production might occur as a result of senescence ([39–41], unpublished data). To establish the existence and direction of a causal relationship, we slowed down mitochondrial superoxide production by mild uncoupling in young cells, because mitochondrial uncoupling has been proposed as a mechanism of cellular adaptation to protect against oxidative stress [42].

Treatment of MRC-5 cells with a 250 μM concentration of the uncoupler 2,4-dinitrophenol (DNP) resulted in mild mitochondrial uncoupling as measured by a decreased JC-1 fluorescence ratio (85.2% ± 0.4%, *p* = 0.05; Figure S4A). This is important because severe uncoupling, for instance by overexpression of UCP-1 [43] or by using FCCP and DNP under conditions of high oxygen supply (unpublished data), can increase mitochondrial ROS production. Cellular ATP levels were decreased following DNP treatment and reached 75% ± 10% of those in untreated cells after 2 wk. Moreover, long-term DNP treatment prevented the further increase of mitochondrial mass that is characteristic for cells approaching senescence (Figure S4B).

**Figure 3:**
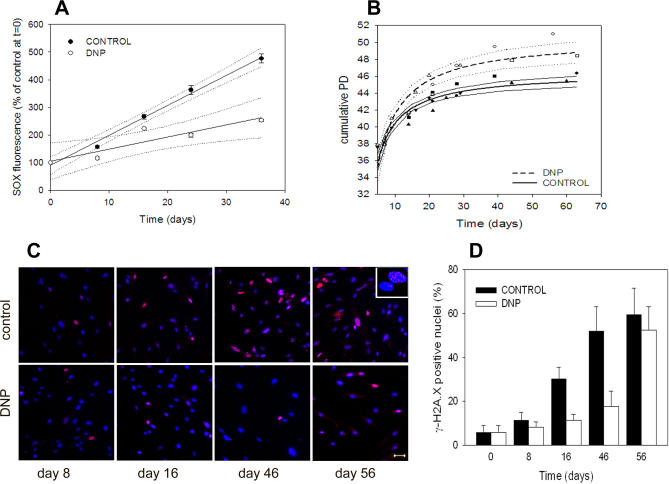
Mild Uncoupling by DNP Prolongs Replicative Lifespan of Human Fibroblasts MRC5 fibroblasts were treated with 250 μM DNP from PD 35 till senescence. (A) MitoSOX fluorescence. Data are mean + Δm, *n* = 2. Fluorescence intensity at *t* = 0 was set to 100%. Solid line: linear regression, dotted lines: 95% confidence intervals. (B) Replicative lifespan. Data are from three (DNP) respective four (control) independent experiments. Hyperbolic best fits and 95% confidence intervals are shown. (C) γ-H2A.X staining. The bar denotes 20 μm. Insert: higher magnification showing focal staining. (D) Frequency of γ-H2A.X-positive fibroblasts. Data are mean ± s.d., *n* = 3. Differences are significant (*p* < 0.05) between day 8 and 46.

Importantly, uncoupling by DNP slowed the accumulation of superoxide in mitochondria of intact fibroblasts (Figure 3A). In comparison to controls, the replicative lifespan was extended by 4–5 PD, amounting to an increase of about 50% of the treated period (Figure 3B). Increased activity of Sen-β-Gal has been associated with cellular senescence [44], and this increase is also delayed under treatment with DNP (Figure S5).

To establish the mechanism by which uncoupling extends replicative lifespan, we first measured the frequency of cells with nuclear foci containing the phosphorylated form of the histone variant H2A.X, γ-H2A.X (Figure 3C). Formation of foci containing γ-H2A.X can be initiated by DNA double-strand breaks or uncapped telomeres, and is a hallmark of the signalling pathway leading to senescence [8,45–47]. Mitochondrial uncoupling delayed foci formation in the majority of cells (Figure 3D).

We next investigated the effect of mitochondrial ROS production on telomere maintenance. Telomere shortening is the major route to telomere uncapping in senescing fibroblasts [9], and the rate of telomere loss in telomerase-negative human somatic cells can be significantly modulated by either increasing oxidative stress [10] or by antioxidant protection [34,37]. However, no direct effect on telomere maintenance has been demonstrated before.

**Figure 4:**
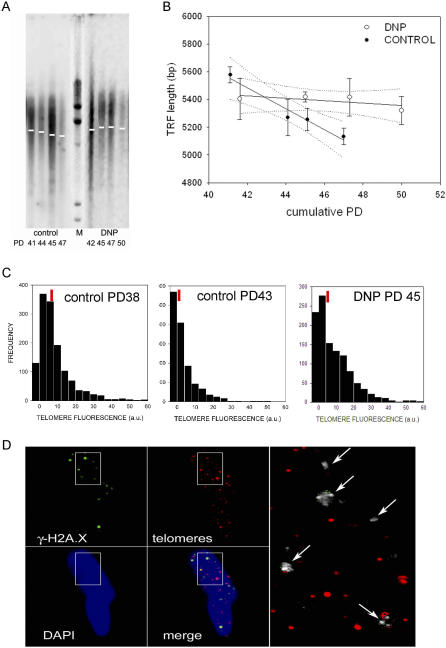
Delayed Senescence in DNP-Treated Fibroblasts Is Telomere Dependent (A) Telomere in-gel hybridisation of untreated (control) and DNP-treated MRC5. The size-weighted average of each peak is indicated by a white line. A λHindIII DNA digest is used as size marker (M). (B) Average telomere (TRF) length in controls and DNP-treated MRC5 fibroblasts. Data are mean ± s.e.m. from quadruplicate gels. Solid line: linear regression, dotted lines: 95% confidence intervals. Differences between slopes are significant (2-tailed *t* test, *p* = 0.002). (C) Telomere Q-FISH histograms of untreated (control) and DNP-treated MRC5 at the indicated PD. Medians are indicated by red bars and are at 6.0 arbitrary units (a.u.) (control PD 38), 1.3 a.u. (control PD 43), and 5.7 a.u. (DNP PD 45). Histograms of control PD 43 and DNP PD 45 are significantly different from each other according to a Mann-Whitney test (*p*-value < 2.2 × 10^−16^). (D) ImmunoFISH of senescent DNP-treated cells at PD 50. γ-H2A.X–containing foci are shown in green and telomeres in red. The boxed area is shown as deconvoluted image at higher magnification at the right. Pixels that show significant co-localisation between foci and telomeres according to a Pearson correlation analysis are shown in white (indicated by arrows).

Mitochondrial uncoupling by DNP resulted in improved telomere maintenance (Figure 4A). The telomere shortening rate of 80 ± 14 base pairs (bp)/PD in controls decreased to 9 ± 29 bp/PD in treated cells, so that DNP-treated cells senesced with longer telomeres than controls (Figure 4B). This effect of DNP was confirmed by measuring telomere length by quantitative fluorescence in situ hybridisation (Q-FISH) on metaphases (Figure 4C). The telomere shortening rate measured here in DNP-treated cells is lower than what would be expected if telomere shortening was governed by simple overhang resection [48]. However, overhang length and telomere shortening are not correlated in human fibroblasts [49]. Moreover, telomere shortening was similarly slow in other fibroblast systems under comparatively low oxidative stress [12,33,50]. To further corroborate the association between oxidative stress levels and telomere shortening, ROS levels in three different human fibroblast strains were modified by growth under high or low oxygen concentrations, treatment with the free radical scavenger PBN, or overexpression of SOD, and telomere shortening was measured by two independent methods (in-gel hybridisation and real-time PCR). A strong positive correlation was obtained (Figure S6), suggesting that lowering the levels of ROS is relevant for the decreased telomere shortening rate under DNP treatment.

Because DNP-treated cells (Figure 4B and 4C), similar to cells treated with PBN [33], senesced with longer telomeres as controls, we next wanted to evaluate the extent of co-localisation between telomeres and DNA-damage foci. There was evident co-localisation between telomeres and γ-H2A.X–containing foci in DNP-treated cells at senescence (Figure 4D). Quantitative evaluation of the deconvoluted images confirmed significant correlation between telomeres and foci equal to the extent of co-localisation seen in senescent control fibroblasts (see below), indicating that the majority of these are telomere dysfunction-induced foci (TIFs). This suggests the possibility that lowering cellular ROS levels might increase the probability of telomere uncapping. Alternatively, there might also an as yet unknown telomere-independent component of lowering ROS levels upon senescence.

Together, these data indicate that mitochondrial uncoupling delayed replicative senescence by slowing down oxidative stress-dependent telomere shortening.

### Variation of Mitochondrial ROS Production Accounts for Cell-to-Cell Heterogeneity in Telomere-Dependent Replicative Lifespan

**Figure 5:**
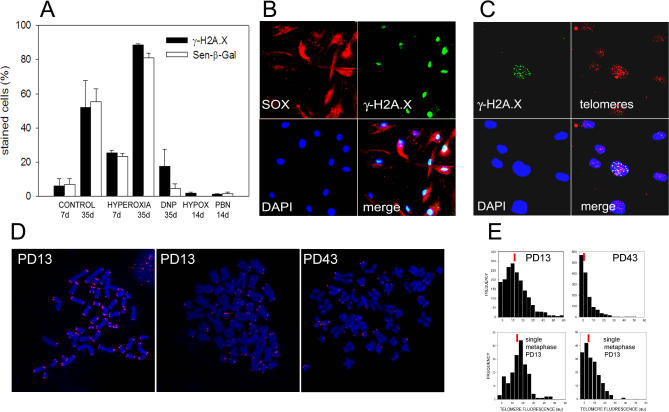
Cell-to-Cell Heterogeneity in Replicative Senescence: Involvement of Mitochondrial ROS and Telomeres (A) The frequency of senescent cells as measured by γ-H2A.X (filled bars) or Sen-β-Gal (open bars) staining is time- and oxidative stress–dependent. Experiments started at PD 38, and cells were grown for the indicated time under either 21% oxygen (CONTROL), 40% normobaric oxygen (HYPEROXIA), normoxia + 250 μM DNP (DNP), 5% normobaric oxygen (HYPOX), or normoxia and 400 μM PBN (PBN). Data are mean ± s.e.m., *n* ≥ 3. (B) Cells with high mitochondrial superoxide production are γ-H2AX positive. Cells were stained with MitoSOX, photographed, and then fixed and immunostained for γ-H2A.X; then the same area was photographed again and merged. (C) ImmunoFISH in an actively growing MRC5 culture shows frequent co-localization (yellow) between γ-H2A.X foci (green) and telomeres (red) in those cells that do contain foci. This co-localisation is significant according to a Pearson correlation analysis (see Figure 6H). (D) Telomere Q-FISH of metaphases from young (left and middle) and near-senescent (right) MRC5 cultures. A few metaphases from young cultures show weak telomeric signals (middle image) similar to typical metaphases from a near-senescent culture (right image). (E) Cumulative Q-FISH telomere frequency histograms from a young (PD 13, top left) and a near-senescent (PD 43, top right) population together with histograms from two individual metaphases at PD 13 (bottom). Metaphases of the near-senescent type (bottom right) are found with low frequency (<10%) in young cultures.

Having established that, in human fibroblasts, (1) mitochondrial dysfunction is associated with replicative senescence, (2) such dysfunction induces a phenotype akin to retrograde response, (3) mitochondrial dysfunction and retrograde response are linked to mitochondrial uncoupling, and (4) uncoupling modulates stress-dependent telomere shortening, we were able to test whether the intrinsically stochastic nature of ROS production and mitochondrial dysfunction could account for the intrinsic heterogeneity of replicative senescence. Around 5%–10% of early passage fibroblasts appear senescent as indicated by various biomarkers [51,52] (Figure 5A). Thus, lifespan heterogeneity can be characterized by the fraction of cells displaying a senescent phenotype.

**Figure 6:**
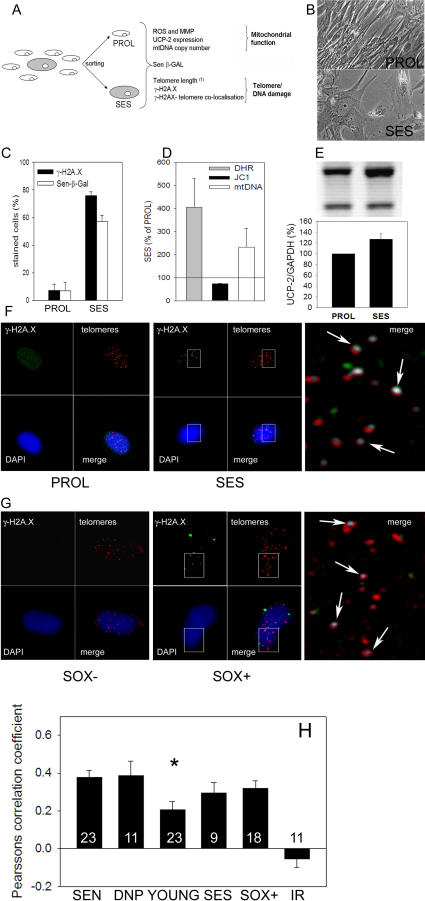
Separation of Cells with a Senescent Phenotype out of Proliferating Cultures Shows Mitochondrial Dysfunction and TIFs (A) Outline of the sorting experiments. Senescent (SES) and proliferating (PROL) cells were sorted by FACS out of growing MRC5 fibroblast cultures according to size and lipofuscin content as described [51], then replated, and the indicated parameters were measured. (B) Morphology of sorted cells 1 wk after replating. (C) Frequency of senescent cells as measured by γ-H2A.X (filled bars) or Sen-β-Gal (open bars) staining in SES- and PROL-sorted MRC5 fibroblasts. Data are mean ± s.e.m., *n* = 3. (D) DHR and JC-1 ratio intensities and mtDNA copy numbers in SES cells relative to those in PROL-sorted fibroblasts, mean ± s.e.m., *n* ≥ 3. (E) Duplex-RT-PCR of UCP-2 versus GAPDH in SES- and PROL-sorted MRC5 fibroblasts (top) and quantification of results (bottom). Data are mean ± s.e.m., *n* = 3. (F) Absence of γ-H2A.X staining in PROL cells and co-localisation of γ-H2A.X foci (green) with telomeres (red) in SES cells. Pixels that show significant co-localisation between foci and telomeres according to a Pearson correlation analysis are shown in white (indicated by arrows) in the magnified image on the right. (G) Absence of γ-H2A.X staining in cells sorted for low MitoSOX fluorescence intensity (SOX-) and co-localisation of foci (green) with telomeres (red) in cells with high MitoSOX fluorescence (SOX+). Magnified image on the right shows significant co-localisation as white pixels (indicated by arrows). (H) Average foci-telomere Pearson correlation coefficients for cells in senescent culture (SEN), DNP-treated senescent cells (DNP), cells that contain γ-H2A.X foci in young culture (YOUNG), SES cells, cells sorted for high MitoSOX staining intensity (SOX+), and cells 48 h after 20-Gy γ-irradiation (IR) as negative control. All conditions are significantly different from the negative control, and the asterisk (*) marks a significant difference to SEN (ANOVA). Data are mean ± s.e.m., with the number of cells evaluated given for each condition.

We noted first that conditions modulating mitochondrial ROS generation significantly altered the frequency of senescent cells. Increasing ambient oxygen led to a time-dependent increase of the fraction of γ-H2A.X– or Sen-β-Gal–positive cells, whereas reduction of ambient oxygen, treatment with the free radical scavenger PBN, or mild mitochondrial uncoupling all decreased this fraction (Figure 5A). Confocal microscopy also revealed considerable cell-to-cell heterogeneity of mitochondrial superoxide levels and membrane potential among cells in proliferating culture (Figure 1A and 1B).

To test the hypothesis that cells with higher mitochondrial ROS production might be the ones to develop earlier senescence, we stained a proliferating fibroblast culture sequentially for MitoSOX and γ-H2A.X. This showed that in the vast majority of cases, cells with dysfunctional mitochondria harboured many nuclear γ-H2A.X foci (Figure 5B).

We next examined the extent of co-localisation between γ-H2A.X foci and telomeres in foci-positive cells at early passage (Figure 5C). In these cells, foci and telomeres were significantly correlated; however, the correlation coefficient was lower than in senescent cells (Figure 6H). Given that some of the foci-containing cells at early passage are not senescent, but S phase cells displaying ATR-dependent foci at replication forks [53], this result confirms the idea that early senescence in young cultures is telomere dependent. Accordingly, by analysing metaphases from early passage HDFs by telomere Q-FISH (Figure 5D), we found that some of these metaphases displayed telomere length distributions very similar to those in metaphases from near-senescent cultures (Figure 5D and 5E).

Together, data shown in Figure 5 provide evidence linking mitochondrial dysfunction and superoxide production to telomere loss and induction of senescence via TIF formation.

To obtain more direct proof for the linkage between mitochondrial dysfunction, telomere maintenance, and early senescence, we used fluorescence-activated cell sorting (FACS) to physically sort cells with a senescent phenotype (SES cells) out of early passage using cell size and lipofuscin content (autofluorescence) as sorting parameters, because both are strongly and positively associated with human fibroblast senescence [51,54,55] (see Figure 6A). SES cells sorted for large size and high lipofuscin content did not grow when replated, but showed the typical morphology of senescent fibroblasts (Figure 6B). On the contrary, cells sorted for small size and low lipofuscin content grew actively (PROL cells; Figure 6B). Staining of PROL and SES cells for the senescence marker Sen-β-Gal and γ-H2A.X confirmed a high degree of enrichment (Figure 6C). SES cells had significantly higher DHR fluorescence (407.0% ± 125% *p* = 0.05) and lower JC-1 fluorescence ratios (63.0% ± 10.7% *p* = 0.03) than the proliferating population (Figure 6D), and showed higher expression levels of UCP-2 (Figure 6E). They also displayed higher mtDNA copy number (233% ± 82%, *n* = 4, *p* = 0.029, Figure 6D). The increase in mtDNA damage in SES cells did not reach statistical significance (unpublished data). Together, these data indicate mitochondrial dysfunction in SES cells similar to that found in a senescent population.

All analysed SES cells showed γ-H2A.X foci, and many of these co-localised with telomeres, whereas there were hardly any nuclei containing foci in PROL cells (Figure 6F). To confirm the role of mitochondrial superoxide generation for telomere-dependent early senescence, we also sorted fibroblasts in early passage according to their MitoSOX fluorescence intensity. There were no γ-H2A.X–positive cells in the low-MitoSOX population, whereas all analysed cells in the high-MitoSOX group showed nuclear γ-H2A.X foci, with the majority of these containing TIFs (Figure 6G). Quantitative correlation analysis again showed the same degree of foci-telomere co-localisation as in senescent cultures (Figure 6H). Thus, cells separated according to either large size and high lipofuscin content or high mitochondrial superoxide production show the same frequency of TIFs as senescent cells.

## Discussion

Since the pioneering work of Harman [56,57], a wealth of data has been accumulated that supports a causal role for mitochondrial dysfunction and ROS production in aging of postmitotic cells [26]. Mitochondrial dysfunction induces retrograde response, a pathway that signals electron transport chain disruption to the nucleus, thus causing wide-ranging adaptations. This is an important part of replicative aging in yeast [58,59]. Interestingly, induction of the retrograde response plays partially contradictory roles in yeast aging: as a part of the normal aging process, it can extend lifespan if activated early, but it also contributes to genomic instability and thus curtails longevity [58].

Retrograde signalling has been described in human cells deprived of mitochondrial DNA or following mitochondrial uncoupling [30,60]. Disruption of the MMP results in elevated cytoplasmic free [Ca^2+^]_i_ and activation of Ca^2+^-dependent signalling including calcineurin and the NFkB pathway, PKC, CREB, and the JNK/MAPK pathway. Retrograde signalling includes up-regulation of mitochondrial biosynthesis via PGC-1 and PPAR-γ, major metabolic and anti-apoptotic adjustments, and possible interaction with TOR signalling [27]. However, there appears to be a large amount of plasticity in this response, depending on, among other things, the specific treatment and cell line examined. Moreover, the role, if any, of mitochondrial dysfunction and retrograde signalling in mammalian cellular senescence is poorly understood [25].

Our data showing decreasing mitochondrial membrane potential, increased mitochondrial biogenesis, decreased capacity for cytoplasmic [Ca^2+^]_i_ regulation, up-regulation of various genes involved in Ca^2+^-dependent signalling pathways, metabolic readjustment, and activated anti-apoptotic response are consistent with induction of retrograde signalling in near-senescent human fibroblasts. It should be stressed that the specific subset of genes identified in Figure 1G–1K cannot be taken as an unbiased representation of retrograde response, because they haven been isolated a posteriori from a sample of differentially expressed genes, and they have not been shown to be individually related to this process. However, they might represent a first approach towards candidates for “signature” genes of retrograde response in senescent fibroblasts. Our data are in accord with recent data showing metabolic impairment in senescent cells, including a significant decline in ATP levels [61]. Thus, we propose that mitochondrial dysfunction and resultant retrograde signalling might be a conserved mechanism in cellular replicative aging from yeast to man.

Interestingly, our results suggest that the disruption of the MMP in senescent fibroblasts is not simply the result of direct ROS-mediated damage. Rather, it is at least in part an adaptation mediated by increased expression of UCP-2 and, possibly, UCP-3. Low MMP together with high ROS levels was found before in postmitotic cells [62]. Activity of uncoupling proteins, including UCP-2, is induced in response to lipid peroxidation products [63] or to superoxide anion in vitro [32] and in vivo [64]. UCP-2 increases the proton leak and decreases MMP and mitochondrial ROS production [65]. This led to the hypothesis of mitochondrial uncoupling as an adaptive mechanism to oxidative stress with potential relevance for aging of postmitotic cells [42]. However, this hypothesis has recently been challenged by showing that physiologically controlled overexpression of SOD-2 in mice, although resulting in decreased mitochondrial superoxide production, did not alter mitochondrial coupling, MMP, or activity of uncoupling proteins in brown fat or skeletal mitochondria [66]. There are conflicting data regarding the interaction between mitochondrial uncoupling and ROS generation and its role for aging in vivo: Mitochondrial uncoupling levels correlated positively with lifespan in outbred mice [67], and overexpression of the human UCP-2 in adult neurons decreased cellular oxidative damage and extended the lifespan of flies [68]. However, mitochondrial coupling decreased with age in mice skeletal muscle concomitant with altered cellular metabolism and energetics, but without observable changes in the expression of UCP-3, the major uncoupling protein in muscle [69]. Moreover, long-term caloric restriction increased rat skeletal muscle UCP-3 content and decreased mitochondrial H_2_O_2_ production, but did not increase leak-dependent (state 4) respiration [70]. Thus, a thorough study of the effects of ROS level variation on the expression of uncoupling proteins and MMP in a well-defined cellular system was warranted.

We found here significant inverse correlations between both mitochondrial superoxide and cellular peroxide levels and MMP, which were independent of the specific type of intervention used to modify ROS levels. This confirms a primary signalling role for ROS in the regulation of mitochondrial uncoupling in human fibroblasts. Senescent and SES fibroblasts both had high ROS levels, high expression of UCP-2, and an MMP as predicted by the ROS intervention experiments (see Figure 2). This suggests that up-regulation of UCP-2, mitochondrial uncoupling, and retrograde response are part of an integral adaptation process of senescent fibroblasts to high ROS levels. Supporting this is the finding that knock-down of UCP-2 generates higher MMP and higher superoxide production in young cells.

The association between mitochondrial dysfunction and senescence might be because mitochondrial dysfunction occurs after the cells have exited the cell cycle. However, reversible quiescent G0 arrest in young cultures did not lead to similar effects (unpublished data). More important, artificial mild mitochondrial uncoupling by DNP not only improved telomere length maintenance, slowed down the rate of TIF formation, and elongated lifespan, but it also diminished the increase in mitochondrial ROS production and slowed down mitochondrial biogenesis, one major component of the retrograde response. Thus, the data strongly suggest that MMP-dependent ROS production is a determinant of telomere-dependent replicative senescence.

This argument is strengthened by the observation of intra-clonal variation in replicative lifespan of human fibroblasts, which indicates that stochastic factors must determine the potential for each individual cell to divide [7]. This intra-clonal heterogeneity in lifespan has been explained both by theoretical models involving mitochondrial ROS production and telomere shortening [21], and experimental work showing shorter telomeres in SES cells as compared to the proliferating ones in the same clone [51]. However, so far, the causal chain leading to variable telomere shortening in individual cells has not been elucidated. Heterogeneity in telomere length might simply be a result of intrinsic variation in leading/lagging strand replication and/or telomere end processing. However, our data show that cell-to-cell heterogeneity is governed by the levels of oxidative stress, both at the population level (Figure 5A) and at the single-cell level (Figure 5B). Sorting cells with a senescent phenotype out of early passage cultures, we observed the same differences as between senescent and young cells in terms of mitochondrial superoxide production, UCP-2 levels, and MMP, indicating that cell-to-cell variation in the degree of mitochondrial dysfunction and mitochondrial ROS production determines heterogeneity of cellular senescence. In accordance with earlier data [10,71] and with those presented above regarding the ROS-dependency of replicative senescence, ROS level variation is a significant determinant of variable telomere shortening, both in terms of average telomere length (Figure 4A) and of telomere length at individual chromosomes (Figure 5D and 5E).

Thus, we suggest that stochastic variation, occurring at the level of mitochondrial ROS production, mitochondrial dysfunction, and retrograde response, determines the probability of telomere uncapping and thus, cell-to-cell heterogeneity of replicative senescence. Further work is necessary to decide whether mtDNA damage is a cause or a consequence of increased mitochondrial ROS production.

## Materials and Methods

### Cells.

MRC-5 human embryonic lung fibroblasts were from ECACC and P43, and P100 fibroblasts were obtained from adult skin [34]. Cells were grown in Dulbecco's Modified Eagle's Medium (DMEM; Sigma, http://www.sigmaaldrich.com) plus 10% fetal calf serum (Sigma). Ambient oxygen partial pressure was modified using 3-gas cell culture incubators (Zapf Instruments, Sarstedt, Germany) to either 95% air, 5% CO_2_ (termed *normoxia*), 40% O_2_, 5% CO_2_ (termed *hyperoxia*), or 5% O2, 5% CO_2_ (termed *hypoxia*). Early senescent cells were sorted by FACS out of young MRC-5 cultures as the quartiles of cells with the highest forward scatter (for size) and FL1 values (for lipofuscin autofluorescence) as described [51]. Low- and high-MitoSOX cells were sorted in a Becton-Dickinson FACSort (http://www.bdbiosciences.com) as the quartiles of cells with the highest and lowest FL3 values following MitoSOX staining. MRC-5 cells at a PD level of 25–30 were transiently transduced with either of three siRNAs specific for human UCP-2 (si1-UCP: 5′-GCCUGUAUGAUUCUGUCAATT-3′, si2-UCP: 5′-CCUGUAUGAUUCUGUCAAATT-3′, and si3-UCP: 5′-CCCUUACCAUGCUCCAGAATT-3′) or a control siRNA (scrambled UCP2 sequence). Cells were assayed 72 h after transfection.

### ROS levels, mitochondrial membrane potential, and mitochondrial mass.

To measure mitochondrial superoxide, cells were stained in 5 μM MitoSOX Red (Molecular Probes, http://www.invitrogen.com) for 10 min at 37 °C, and FL3 median fluorescence intensity was measured by flow cytometry (Partec PAS, http://www.partec.com). Specificity of MitoSOX for superoxide has been shown by the manufacturer, and its mitochondrial localisation was tested by co-staining with Mitotracker Green (unpublished data). Cellular peroxide levels were assessed by staining with 30 μM DHR (Molecular Probes) for 30 min at 37 °C and analysis of FL1 fluorescence. As a positive control, cells were treated with H_2_O_2_ (0 to 400 μM for 30 min) before DHR staining (unpublished data). MMP was measured as the FL3/FL1 ratio after staining cells with 1-μg/ml JC-1 (5,5',6,6'-tetrachloro-1,1',3,3'- tetraethylbenzimidazolylcarbocyanine iodide; Molecular Probes) in phenol-free RPMI 1640 (Sigma) for 30 min at 37 °C. Uncoupling with carbonyl cyanide p-trifluoromethoxyphenylhydrazone (FCCP) (20 μM for 30 min) was used as positive control, resulting in a 60% decrease of the JC1 ratio. Mitochondrial mass was measured as FL1 fluorescence after staining of cells with 10 μM nonyl acridine orange (NAO; Molecular Probes) for 10 min at 37 °C in the dark. The flow cytometer was calibrated using fluorescent microspheres. All data are mean ± s.e.m. from *n* independent experiments with measurements in duplicate and 10^4^ cells per measurement.

Cells growing on coverslips were stained as above and observed using a Zeiss LSM 510 Meta confocal microscope (Carl Zeiss, http://www.zeiss.com). Conventional transmission electron microscopy was used to confirm NAO staining. Twelve sections of cells with central nuclei per group were systematically random sampled to measure the volume density of mitochondria per cell.

### Intracellular calcium imaging.

Twenty-four hours prior to imaging, cells were plated in DeltaT dishes (Bioptechs, http://www.bioptechs.com) at a density of 50,000 cells. Fluo3-AM (Molecular Probes) calcium dye (1 μM) was loaded for 30 min then washed three times in medium. Fluorescence imaging was performed at room temperature using an LSM 510 Meta confocal microscope. Calcium concentrations were determined as described by [72]. Basal [Ca^2+^]_i_ was determined from the average of ten consecutive images for each cell. Images were obtained from three independent experiments. Recovery to basal [Ca^2+^]_i_ was determined as the time to reach 1/*e* from the maximal response to addition of exogenous 100 mM CaCl_2_.

### Telomere length.

Telomere restriction fragment length was measured by in-gel hybridisation following pulsed-field gel electrophoresis (CHEF3; BioRad, http://www.bio-rad.com) as described [51]. The amount of loaded DNA and its integrity was controlled by EtBr staining of total DNA in the gel and by re-hybridisation with a (CAC)_8_ probe as described [34]. The average telomere length was calculated as weighted mean of the peak using AIDA v3.11 image analysis software (Raytek, http://www.raytest.de). In some experiments, telomere length was additionally measured using real-time PCR as described [51]. The telomere shortening rate per PD was calculated as the slope of the linear regression between telomere length and PD.

### γ-H2A.X immunostaining.

Cells grown on coverslips were fixed in 2% paraformaldehyde and incubated with mouse monoclonal anti-γ-H2A.X (Upstate Biotechnology, http://www.upstate.com) for 1 h at room temperature. Slides were analysed in a Zeiss LSM 510 Meta confocal microscope. For MitoSOX/γ-H2A.X co-staining, cells were grown on gridded coverslips and first stained with MitoSOX as described above. Cells were mounted in 50% glycerol, photographed by confocal microscopy, and then immunostained for γ-H2A.X. The same areas of the grid were identified and images for γ-H2A.X taken.

### Q-FISH.

Metaphases were prepared by treatment of subconfluent cells with 10-μg/ml Colcemid for 2 h at 37 °C, followed by 60 mM KCl for 15 min at room temperature and fixation in ethanol: acetic acid (3:1). Slides were air dried and baked at 60 °C for 1 h, re-hydrated in 1 ml of 2× SSC at 37 °C for 2 min, and dehydrated. A total of 20 μl of Cy-3–labelled telomere-specific (C_3_TA_2_)_3_ peptide nucleic acid (PNA) probe (4 ng/μl) (DakoCytomation, http://www.dako.com) was applied to the cells followed by co-denaturation at 85 °C and hybridisation for 2 h at room temperature in the dark. Cells were washed with 2× SSC/0.05% Tween for 10 min at room temperature. Telomere signals from at least 14 metaphases per group were quantified using TelomereQuant v 1.0 (Dako).

### ImmunoFISH.

Cells grown on coverslips were fixed, and γ-H2A.X immunostaining was performed as described above. After application of the secondary antibody, cells were washed with PBS, and Q-FISH was performed as described. After washing with SSC/0.5% Tween for 10 min, the secondary antibody incubation was repeated, followed by DAPI staining, mounting, and imaging using a Zeiss LSM 510 Meta confocal microscope. For co-localisation analysis, confocal images were deconvolved and analysed using Costes approximation method [73] in ImageJ, v1.37a (http://rsb.info.nih.gov/ij/). For each nucleus, identified by DAPI staining, a Pearson's correlation coefficient *(R)* was determined between the fluorescein isothiocyanate (FITC) and Cy3 images (demonstrating the amount of co-localisation between the two fluorescent images on a scale of +1 to −1, representing perfect co-localisation to no co-localisation, respectively). The co-localised pixels obtained in the co-localisation analysis were represented in a third image as a white pixel overlay (Figures 4D, 6F, and 6G).

## Supporting Information

Figure S1 Senescent MRC5 Fibroblasts Contain More Mitochondria Than Young Ones (A) Electron micrographs of perinuclear areas in proliferating (PD 35, left) and near-senescent (PD 46, right) cells. Note the perinuclear accumulation and degenerative changes of mitochondria, such as formation of cup-shaped (c) and circular mitochondria containing inclusions of dense osmiophilic material, in the senescent cell. n, nucleus; m, mitochondrion.
(B) Confocal images of NAO-stained fibroblasts taken under identical imaging conditions. Note the intense perinuclear staining in the senescent cell.
(C) Mitochondrial volume density V_V_ mitochondria (in percentage of cell volume) as measured by electron microscopic morphometry. Data are mean ± s.e.m. from 12 cells per group.
(D) Quantification of NAO fluorescence by FACS, indicating mitochondrial mass per cell. Data are mean ± s.e.m., *n* = 4.
(E) Relative mtDNA copy number as measured by quantitative real-time PCR. Data are mean ± s.e.m., *n* = 3.
(426 KB JPG)

Figure S2 Hierarchic Cluster Analysis of Gene Expression in Senescent MRC5 Cells Young fibroblasts arrested by confluency in G0/G1 (four independent experiments, YOUNG) were compared to fibroblasts grown to senescence under normoxia (three experiments, SEN). A total of 2,610 probe sets (accession numbers) were changed at least 2-fold between YOUNG and SEN, with the majority (1,552) of these being down-regulated in senescent cells. Only a limited number of these changes were confirmed in fibroblasts grown to senescence under hyperoxia (two experiments, HYPER). Expression levels are colour-coded as shown on the right.
(673 KB JPG)

Figure S3 Up-Regulation of Signalling and Metabolic Enzymes in Senescence and under Hyperoxia RT-PCR was performed for the genes indicated on the right using conditions indicated in Table S1. GAPDH was measured as loading control. H, cells kept under hyperoxia for 47 d; S, senescent cells (PD 48); Y, young cells (PD 25).
(139 KB JPG)

Figure S4 Effect of Long-Term DNP Treatment on MMP and Mitochondrial Mass (A) DNP treatment decreases MMP in pre-senescent, but not in senescent cells. MMP was measured as FL3/FL1 ratio of JC-1–stained cells treated with 250 μM DNP for the indicated times. Data are mean ± s.e.m., *n* = 3. Stars denote significant differences to controls with *p* = 0.05 (Paired *t*-test, two-tailed).
(B) Long-term DNP treatment partially compensates for the increase in mitochondrial mass during senescence. Mitochondrial mass measured as FL1 fluorescence of NAO-stained cells. Data are mean ± s.e.m., *n* = 3. Star denotes significant differences to controls with *p* = 0.003 (Paired *t*-test, two-tailed).
(147 KB JPG)

Figure S5 DNP Slows Down the Accumulation of Sen-β-Gal–Positive Cells Control cells at PD 46 (A) and DNP-treated cells at PD 50 (B) were both stained for Sen-β-Gal at day 56 of the experiment.
(237 KB JPG)

Figure S6 Correlation between Cellular ROS Levels and Telomere Shortening Rates in Human Fibroblasts ROS levels were varied in three human fibroblast strains (P43, upward triangles; P100, downward triangles; and MRC5, circles) by growing cells until senescence under either normoxia (filled symbols), hyperoxia (open symbols), hypoxia (red symbols), or hypoxia plus addition of 400 μM α-phenyl-butyl-nitrone (PBN), a free radical scavenger (pink symbols). MRC5 cells were not grown under hypoxia, but were treated with 250 μM DNP (green symbol) or were transfected with an SOD3 transgene [34] (yellow symbol) instead. Cytoplasmic peroxide levels were measured by DHR flow cytometry. DNA samples were taken every 2–3 PD (a total of between three and 14 samples per strain and condition), and telomere length was measured by in-gel hybridisation and (for P100 cells) by real-time PCR. Measurements were performed in quadruplicate gels or PCRs, and telomere shortening rates were calculated by linear regression. Data are mean ± s.e.m. The correlation between DHR fluorescence and telomere shortening rate was modelled by an exponential regression with an *r* ^2^ = 0.956.
(171 KB JPG)

Protocol S1 Supplemental Protocols (35 KB DOC)

Table S1 PCR Conditions (28 KB DOC)

Table S2 KEGG Pathway Functional Enrichment Analysis: 20 Most Statistically Significant Annotation Terms (42 KB DOC)

## Abbreviations

DHR: dihydrorhodamine
FACS: fluorescence-activated cell sorting
HDF: human diploid fibroblast
MitoSOX: mitochondrial superoxide indicator
MMP: mitochondrial membrane potential
mtDNA: mitochondrial DNA
PD: population doublings
Q-FISH: quantitative fluorescence in situ hybridisation
ROS: reactive oxygen species
RT-PCR: reverse-transcriptase PCR
s.e.m.: standard error of the mean
Sen-β-Gal: senescence-associated β-galactosidase
SES: sorted early senescent
siRNA: small interfering RNA
TIF: telomere dysfunction-induced focus

## References

[pbio-0050110-b001] Kirkwood TBL (2005). Understanding the odd science of aging. Cell.

[pbio-0050110-b002] Finch CE, Kirkwood TBL (2000). Chance, development and aging.

[pbio-0050110-b003] Herndon LA, Schmeissner PJ, Dudaronek JM, Brown PA, Listner KM (2002). Stochastic and genetic factors influence tissue-specific decline in ageing C. elegans. Nature.

[pbio-0050110-b004] Rea SL, Wu D, Cypser JR, Vaupel JW, Johnson TE (2005). A stress-sensitive reporter predicts longevity in isogenic populations of Caenorhabditis elegans. Nat Genet.

[pbio-0050110-b005] Bahar R, Hartmann CH, Rodriguez KA, Denny AD, Busuttil RA (2006). Increased cell-to-cell variation in gene expression in ageing mouse heart. Nature.

[pbio-0050110-b006] Hayflick L, Moorhead PS (1961). The serial cultivation of human diploid cell strains. Exp Cell Res.

[pbio-0050110-b007] Smith JR, Whitney RG (1980). Intraclonal variation in proliferative potential of human diploid fibroblasts: Stochastic mechanism for cellular aging. Science.

[pbio-0050110-b008] D'Adda di Fagagna F, Reaper PM, Clay-Farrace L, Fiegler H, Carr P (2003). A DNA damage checkpoint response in telomere-initiated senescence. Nature.

[pbio-0050110-b009] Blackburn EH, Chan S, Chang J, Fulton TB, Krauskopf A (2000). Molecular manifestations and molecular determinants of telomere capping. Cold Spring Harb Symp Quant Biol.

[pbio-0050110-b010] von Zglinicki T (2002). Oxidative stress shortens telomeres. Trends Biochem Sci.

[pbio-0050110-b011] Petersen S, Saretzki G, von Zglinicki T (1998). Preferential accumulation of single-stranded regions in telomeres of human fibroblasts. Exp Cell Res.

[pbio-0050110-b012] Sitte N, Saretzki G, von Zglinicki T (1998). Accelerated telomere shortening in fibroblasts after extended periods of confluency. Free Rad Biol Med.

[pbio-0050110-b013] Campisi J (2005). Senescent cells, tumor suppression, and organismal aging: Good citizens, bad neighbors. Cell.

[pbio-0050110-b014] Collado M, Gil J, Efeyan A, Guerra C, Schuhmacher AJ (2005). Tumour biology: Senescence in premalignant tumours. Nature.

[pbio-0050110-b015] Braig M, Lee S, Loddenkemper C, Rudolph C, Peters AHFM (2005). Oncogene-induced senescence as an initial barrier in lymphoma development. Nature.

[pbio-0050110-b016] Michaloglou C, Vredeveld LCW, Soengas MS, Denoyelle C, Kuilman T (2005). BRAFE600-associated senescence-like cell cycle arrest of human naevi. Nature.

[pbio-0050110-b017] Chen Z, Trotman LC, Shaffer D, Lin H-K, Dotan ZA (2005). Crucial role of p53-dependent cellular senescence in suppression of Pten-deficient tumorigenesis. Nature.

[pbio-0050110-b018] Epel ES, Blackburn EH, Lin J, Dhabhar FS, Adler NE (2004). Accelerated telomere shortening in response to life stress. Proc Natl Acad Sci U S A.

[pbio-0050110-b019] Blackburn EH (2000). Telomere states and cell fates. Nature.

[pbio-0050110-b020] Rubelj I, Vondracek Z (1999). Stochastic mechanism of cellular aging—Abrupt telomere shortening as a model for stochastic nature of cellular aging. J Theor Biol.

[pbio-0050110-b021] Sozou PD, Kirkwood T (2001). A Stochastic model of cell replicative senescence based on telomere shortening, oxidative stress, and somatic mutations in nuclear and mitochondrial DNA. J Theor Biol.

[pbio-0050110-b022] Proctor CJ, Kirkwood TBL (2003). Modelling cellular senescence as a result of telomere state. Aging Cell.

[pbio-0050110-b023] Proctor CJ, Kirkwood TBL (2002). Modelling telomere shortening and the role of oxidative stress. Mech Age Dev.

[pbio-0050110-b024] Taylor RW, Barron MJ, Borthwick GM, Gospel A, Chinnery PF (2003). Mitochondrial DNA mutations in human colonic crypt stem cells. J Clin Inv.

[pbio-0050110-b025] Passos JF, von Zglinicki T (2005). Mitochondria, telomeres and cell senescence. Exp Gerontol.

[pbio-0050110-b026] Balaban RS, Nemoto S, Finkel T (2005). Mitochondria, oxidants, and aging. Cell.

[pbio-0050110-b027] Butow RA, Avadhani NG (2004). Mitochondrial signaling: The retrograde response. Mol Cell.

[pbio-0050110-b028] Kirchman PA, Kim S, Lai C-Y, Jazwinski SM (1999). Interorganelle signaling is a determinant of longevity in Saccharomyces cerevisiae. Genetics.

[pbio-0050110-b029] Miceli MV, Jazwinski SM (2005). Common and cell type-specific responses of human cells to mitochondrial dysfunction. Exp Cell Res.

[pbio-0050110-b030] Biswas G, Adebanjo OA, Freedman BD, Anandatheerthavarada HK, Vijayasarathy C (1999). Retrograde Ca2+ signaling in C2C12 skeletal myocytes in response to mitochondrial genetic and metabolic stress: A novel mode of inter-organelle crosstalk. EMBO J.

[pbio-0050110-b031] Nicholls DG (2004). Mitochondrial membrane potential and aging. Aging Cell.

[pbio-0050110-b032] Echtay KS, Roussel D, St-Pierre J, Jekabsons MB, Cadenas S (2002). Superoxide activates mitochondrial uncoupling proteins. Nature.

[pbio-0050110-b033] von Zglinicki T, Pilger R, Sitte N (2000). Accumulation of single-strand breaks is the major cause of telomere shortening in human fibroblasts. Free Radic Biol Med.

[pbio-0050110-b034] Serra V, von Zglinicki T, Lorenz M, Saretzki G (2003). Extracellular superoxide dismutase is a major antioxidant in human fibroblasts and slows telomere shortening. J Biol Chem.

[pbio-0050110-b035] Packer L, Fuehr K (1977). Low oxygen concentration extends the lifespan of cultured human diploid cells. Nature.

[pbio-0050110-b036] Chen Q, Fischer A, Reagan J, Yan L, Ames B (1995). Oxidative DNA damage and senescence of human diploid fibroblast cells. Proc Natl Acad Sci U S A.

[pbio-0050110-b037] Saretzki G, Murphy MP, von Zglinicki T (2003). MitoQ counteracts telomere shortening and elongates lifespan of fibroblasts under mild oxidative stress. Aging Cell.

[pbio-0050110-b038] von Zglinicki T, Saretzki G, Docke W, Lotze C (1995). Mild hyperoxia shortens telomeres and inhibits proliferation of fibroblasts: A model for senescence?. Exp Cell Res.

[pbio-0050110-b039] Macip S, Igarashi M, Berggren P, Yu J, Lee SW (2003). Influence of induced reactive oxygen species in p53-mediated cell fate decisions. Mol Cell Biol.

[pbio-0050110-b040] Macip S, Igarashi M, Fang L, Chen A, Pan Z (2002). Inhibition of p21-mediated ROS accumulation can rescue p21-induced senescence. EMBO J.

[pbio-0050110-b041] Lee AC, Fenster BE, Ito H, Takeda K, Bae NS (1999). Ras proteins induce senescence by altering the intracellular levels of reactive oxygen species. J Biol Chem.

[pbio-0050110-b042] Brand MD (2000). Uncoupling to survive? The role of mitochondrial inefficiency in ageing. Exp Gerontol.

[pbio-0050110-b043] Bernal-Mizrachi C, Gates AC, Weng S, Imamura T, Knutsen RH (2005). Vascular respiratory uncoupling increases blood pressure and atherosclerosis.

[pbio-0050110-b044] Dimri G, Lee X, Basile G, Acosta M, Scott G (1995). A biomarker that identifies senescent human cells in culture and in aging skin in vivo. Proc Natl Acad Sci U S A.

[pbio-0050110-b045] Takai H, Smogorzewska A, de Lange T (2003). DNA damage foci at dysfunctional telomeres. Curr Biol.

[pbio-0050110-b046] Herbig U, Jobling WA, Chen BPC, Chen DJ, Sedivy JM (2004). Telomere shortening triggers senescence of human cells through a pathway involving ATM, p53, and p21CIP1, but not p16INK4a. Mol Cell.

[pbio-0050110-b047] Zou Y, Sfeir A, Gryaznov SM, Shay JW, Wright WE (2004). Does a sentinel or a subset of short telomeres determine replicative senescence?. Mol Biol Cell.

[pbio-0050110-b048] Huffman KE, Levene SD, Tesmer VM, Shay JW, Wright WE (2000). Telomere shortening is proportional to the size of the G-rich telomeric 3'-overhang. J Biol Chem.

[pbio-0050110-b049] Keys B, Serra V, Saretzki G, von Zglinicki T (2004). Telomere shortening in human fibroblasts is not dependent on the size of the telomeric-3′-overhang. Aging Cell.

[pbio-0050110-b050] Serra V, von Zglinicki T (2002). Human fibroblasts in vitro senesce with a donor-specific telomere length. FEBS Lett.

[pbio-0050110-b051] Martin-Ruiz C, Saretzki G, Petrie J, Ladhoff J, Jeyapalan J (2004). Stochastic variation in telomere shortening rate causes heterogeneity of human fibroblast replicative life span. J Biol Chem.

[pbio-0050110-b052] Kill IR, Faragher RG, Lawrence K, Shall S (1994). The expression of proliferation-dependent antigens during the lifespan of normal and progeroid human fibroblasts in culture. J Cell Sci.

[pbio-0050110-b053] Ward IM, Chen J (2001). Histone H2AX Is phosphorylated in an ATR-dependent manner in response to replicational stress. J Biol Chem.

[pbio-0050110-b054] Bayreuther K, Rodemann HP, Francz PI, Maier K (1988). Differentiation of fibroblast stem cells. J Cell Sci Suppl.

[pbio-0050110-b055] Gorbunova V, Seluanov A, Pereira-Smith OM (2003). Evidence that high telomerase activity may induce a senescent-like growth arrest in human fibroblasts. J Biol Chem.

[pbio-0050110-b056] Harman D (1956). Aging: A theory based on free radical and radiation chemistry. J Gerontol.

[pbio-0050110-b057] Harman D (1972). The biologic clock: The mitochondria?. J Am Geriatr Soc.

[pbio-0050110-b058] Jazwinski SM (2005). The retrograde response links metabolism with stress responses, chromatin-dependent gene activation, and genome stability in yeast aging. Gene.

[pbio-0050110-b059] Nautiyal S, DeRisi JL, Blackburn EH (2002). The genome-wide expression response to telomerase deletion in Saccharomyces cerevisiae. Proc Natl Acad Sci U S A.

[pbio-0050110-b060] Amuthan G, Biswas G, Ananadatheerthavarada HK, Vijayasarathy C, Shephard HM (2002). Mitochondrial stress-induced calcium signaling, phenotypic changes and invasive behavior in human lung carcinoma A549 cells. Oncogene.

[pbio-0050110-b061] Hutter E, Renner K, Pfister G, Stockl P, Jansen-Durr P (2004). Senescence-associated changes in respiration and oxidative phosphorylation in primary human fibroblasts. Biochem J.

[pbio-0050110-b062] Hagen TM, Yowe DL, Bartholomew JC, Wehr CM, Do KL (1997). Mitochondrial decay in hepatocytes from old rats: Membrane potential declines, heterogeneity and oxidants increase. Proc Natl Acad Sci U S A.

[pbio-0050110-b063] Echtay KS, Esteves TC, Pakay JL, Jekabsons MB, Lambert AJ (2003). A signalling role for 4-hydroxy-2-nonenal in regulation of mitochondrial uncoupling. EMBO J.

[pbio-0050110-b064] Krauss S, Zhang C-Y, Scorrano L, Dalgaard LT, St-Pierre J (2003). Superoxide-mediated activation of uncoupling protein 2 causes pancreatic {beta} cell dysfunction. J Clin Invest.

[pbio-0050110-b065] Mattson MP, Liu D (2003). Mitochondrial potassium channels and uncoupling proteins in synaptic plasticity and neuronal cell death. Biochem Biophy Res Comm.

[pbio-0050110-b066] Silva JP, Shabalina IG, Dufour E, Petrovic N, Backlund EC (2005). SOD2 overexpression: Enhanced mitochondrial tolerance but absence of effect on UCP activity. EMBO J.

[pbio-0050110-b067] Speakman JR, Talbot DA, Selman C, Snart S, McLaren JS (2004). Uncoupled and surviving: Individual mice with high metabolism have greater mitochondrial uncoupling and live longer. Aging Cell.

[pbio-0050110-b068] Fridell Y-WC, Sanchez-Blanco A, Silvia BA, Helfand SL (2005). Targeted expression of the human uncoupling protein 2 (hUCP2) to adult neurons extends life span in the fly. Cell Metab.

[pbio-0050110-b069] Marcinek DJ, Schenkman KA, Ciesielski WA, Lee D, Conley KE (2005). Reduced mitochondrial coupling in vivo alters cellular energetics in aged mouse skeletal muscle. J Physiol (Lond).

[pbio-0050110-b070] Bevilacqua L, Ramsey JJ, Hagopian K, Weindruch R, Harper M-E (2005). Long-term caloric restriction increases UCP3 content but decreases proton leak and reactive oxygen species production in rat skeletal muscle mitochondria. Am J Physiol Endocrinol Metab.

[pbio-0050110-b071] von Zglinicki T, Petrie J, Kirkwood TB (2003). Telomere-driven replicative senescence is a stress response. Nat Biotechnol.

[pbio-0050110-b072] Thomas D, Tovey SC, Collins TJ, Bootman MD, Berridge MJ (2000). A comparison of fluorescent Ca2+ indicator properties and their use in measuring elementary and global Ca2+ signals. Cell Calcium.

[pbio-0050110-b073] Costes SV, Daelemans D, Cho EH, Dobbin Z, Pavlakis G (2004). Automatic and quantitative measurement of protein-protein colocalization in live cells. Biophys J.

