# Supplementary material for: Mitochondrial Dysfunction Accounts for the Stochastic Heterogeneity in Telomere-Dependent Senescence
Source: PLoS Biol. 2007 May 1;5(5):e110. doi: 10.1371/journal.pbio.0050110 (PMC1858712; doi:10.1371/journal.pbio.0050110)
Supplement: Supplementary file 1 [file pbio.0050110.sd001.doc]

**Supplemental protocols**

## ATP measurements

ATP in DNP treated cells was determined using a luciferase-based ATP measurement kit (Molecular Probes) according to the manufacturer's instructions with values normalized for total protein.

## Senescence-associated β-Galactosidase Staining

Cells were fixed with 1% formaldehyde in PBS for 5 min and stained as described (Dimri et al., 1995). Cells in 10 randomly chosen fields were counted per experiment.

## Gene expression

Total RNA was extracted from 4 independent early-passage MRC-5 fibroblast cultures that were confluency-arrested in G0, from 3 independent senescent cell cultures and from 2 independent cultures kept for at least 40 days under hyperoxia (40% ambient oxygen) using Trizol (Invitrogen, Paisley, U.K.). Biotin-labeled cRNA was prepared and processed according to the manufacturer’s instructions and analysed using U133 2.0 plus gene chips (Affymetrix, Buckingham, U.K.). First, data from early-passage and senescent cells were compared and functional enrichment of KEGG-defined pathways was assessed using AMDA (Pelizzola et al. 2006). Data from all three conditions were further analysed using GeneSpring v7.0 using the following criteria: at least 4 out of 9 calls marginal or present, average fold change between conditions at least 2fold.

Total RNA was reverse transcribed using Superscript reverse transcriptase (Invitrogen, U.K.). Duplex-PCR for UCP-2 and GAPDH was conducted using a SingleGene PCR Kit (Superarray). PCR conditions for all other genes are given in Tab S1. Cycle numbers were optimized to be in the linear amplification range. Whole cell protein lysates were prepared from young, hyperoxia-exposed and senescent MRC-5 fibroblasts, Western-blotted and probed with rabbit anti-UCP-2 (1:200) (Alpha Diagnostics, San Antonio, TX) or mouse anti-βactin (Sigma) antibodies.

***mtDNA damage and copy number***

mtDNA damage was measured as amplification efficiency for a large (11,095bp) fragment normalized to a short (83bp) amplicon of mtDNA. A published method (Santos et al. 2006) was modified by amplifying both the long and short amplicons in real-time by incorporating SYBR^®^-Green fluorescence. The 83 bp amplicon was amplified using SYBR^®^ Green JumpStart Taq ReadyMix (Sigma) in an MJ Chromo4, using primer nucleotide sequences: 5’-GATTTGGGTACCACCCAAGTATTG-3’ (16042-16064), and 5’-AATATTCATGGTGGCTGGCATGTA-3’ (16125-16102). PCR conditions were as follows: 94°C for 2 mins, followed by cycles of denaturation at 94°C for 30 s, annealing at 60°C for 45 s, and extension at 72°C for 45 s.

The long range PCR was also performed in an MJ Chromo4 using the Expand Long Template PCR system (Roche). A series of dilution standards were prepared for quantification of the products. Reaction mixtures contained ~4x10^7^ mtDNA templates, 2.75 mM Mg^2+^, 0.3 μM primers, 500 μM dNTPs, 0.1x SYBR^®^-Green I (Molecular Probes), and 3.75 units of Expand Taq DNA polymerase. The primer nucleotide sequences were as follows: 5’-GGGAGAAGCCCCGGCAGGTTTGAAGC-3’ (5756-5781), and 5’-ATGATGTCTGTGTGGAAAGTGGCTGTGC-3’ (283-255). PCR conditions were as follows: 94°C for 2 mins, followed by cycles of denaturation at 94°C for 15 s and annealing and extension at 68°C for 8.5 mins with an additional 10 s/cycle. Amplification specificity was confirmed by performing melting curve analyses and visualising PCR products run on an agarose gel. Relative mtDNA copy number was determined by calculating the difference in amplification between the 83 bp mtDNA amplicon and an 84 bp nuclear amplicon in the beta-actin gene using primer nucleotide sequences 5’-CGAGTAAGAGACCATTGTGGCAG-3’, and 5’-GCTGTTCTGTCAATAAATTTCCTTC-3’. The PCR reaction conditions were the same for both.

**Additional references**

Pelizzola, M., Pavelka, N., Foti,M., and P. Ricciardi-Castagnoli, *AMDA: an R package for the automated microarray data analysis.* BMC Bioinformatics 2000, **7**:335.

Santos, J.H., Meyer, J.N, and B. Van Houten*, Quantitative PCR-based measurement of nuclear and mitochondrial DNA damage and repair in mammalian cell*s. Methods Mol Biol, 2006**. 3**14: p. 183-99.
