# Supplementary material for: Mitochondrial Dysfunction Accounts for the Stochastic Heterogeneity in Telomere-Dependent Senescence
Source: PLoS Biol. 2007 May 1;5(5):e110. doi: 10.1371/journal.pbio.0050110 (PMC1858712; doi:10.1371/journal.pbio.0050110)
Supplement: Supplementary file 2 [file pbio.0050110.st001.doc]

Tab S1: PCR conditions

| Gene | Primer sequences | Cycle number | Annealing temperature |
| --- | --- | --- | --- |
| PLCB4 | fw: 5’-gca tcg gga aaa aca gaa aa-3’  rev 5’ gaa aat gct gtg ggc tca at-3’ | 26 | 55 |
| IGFBP3 | fw 5’-ttg cac aaa aga ctg cca ag-3’  rev 5’-caa cat gtg gtg agc att cc-3’ | 26 | 55 |
| TGFβ2 | fw 5’-ccg gag gtg att tcc atc ta-3’  rev 5’-ctc cat tgc tga gac gtc aa-3’ | 26 | 55 |
| MAOA | fw 5’-cac acc ttt tgg gaa atg ct-3’  rev 5’-tgg act ggc att cat ttt ga-3’ | 26 | 50 |
| PDK4 | fw 5’-ctt ttg gct ggt ttt ggt ta-3’  rev 5’-cct gct tgg gat aca cca gt-3’ | 24 | 50 |
| GAPDH | fw 5’-ctcagacaccatggggaaggtga-3’  rev 5’-atgatcttgaggctgttgtcata-3’ | 19 | 55 |
