# Supplementary material for: Mitochondrial Dysfunction Accounts for the Stochastic Heterogeneity in Telomere-Dependent Senescence
Source: PLoS Biol. 2007 May 1;5(5):e110. doi: 10.1371/journal.pbio.0050110 (PMC1858712; doi:10.1371/journal.pbio.0050110)
Supplement: Supplementary file 3 [file pbio.0050110.st002.doc]

**Supplementary table**

**Tab. S2 KEGG pathway functional enrichment analysis: 20 most statistically significant annotation terms**

| ID | Annotation term description | n DEG | n total | p |
| --- | --- | --- | --- | --- |
| 04110 | Cell cycle | 63 | 314 | 7.85 e-19 |
| 04512 | ECM-receptor interactions | 44 | 296 | 2.96 e-09 |
| 04350 | TGF-beta signalling pathway | 39 | 249 | 4.41 e-09 |
| 00230 | Purine metabolism | 51 | 384 | 9.0 e-08 |
| 04060 | Cytokine-cytokine receptor interaction | 63 | 532 | 2.62 e-08 |
| 04630 | JAK-STAT signalling pathway | 49 | 385 | 7.95 e-08 |
| 00910 | Nitrogen metabolism | 15 | 64 | 9.1 e-08 |
| 00251 | Glutamate metabolism | 17 | 91 | 3.85 e-06 |
| 00550 | Peptidoglycan biosynthesis | 3 | 4 | 1.1 e-05 |
| 04340 | Hedgehog signalling pathway | 20 | 132 | 2.23 e-05 |
| 04620 | Toll-like receptor signalling pathway | 28 | 220 | 2.93 e-05 |
| 01430 | Cell communication | 33 | 281 | 3.89 e-05 |
| 04640 | Hematopoetic cell lineage | 26 | 226 | 2.83 e-05 |
| 00920 | Sulphur metabolism | 7 | 33 | 4.56 e-05 |
| 03030 | DNA polymerase | 10 | 60 | 5.68 e-05 |
| 00240 | Pyrimidine metabolism | 22 | 206 | 1.9 e-04 |
| 04010 | MAPK signalling pathway | 68 | 841 | 2.21 e-04 |
| 04310 | WNT signalling pathway | 42 | 476 | 2.79 e-04 |
| 04510 | Focal adhesion | 60 | 736 | 3.06 e-04 |
| 00760 | Nicotinate and nicotinamide metabolism | 19 | 177 | 3.15 e-04 |

ID: KEGG annotation term ID

n DEG: number of differentially expressed genes in category

n total: total number of probesets on the whole chip annotated with the term

p: P value of functional enrichment
